# Supplementary material for: Are carbon emissions trading and green financial instruments synergistic? -Comprehensive quantitative research based on content analysis
Source: PLoS One. 2024 Mar 7;19(3):e0298601. doi: 10.1371/journal.pone.0298601 (PMC10919601; doi:10.1371/journal.pone.0298601)
Supplement: S1 Data — (ZIP) [file pone.0298601.s001.zip › Data/地级市Z_F工作报告.zip!/╣π╬≈/╣≡┴╓╩╨/└┤╫╘╣1⁄2╓┌║┼í╛┬φ┐╦╩2╛▌═°í┐_2016.docx]

各位代表：

　　现在，我代表市人民政府向大会作政府工作报告，请予审议，并请各位政协委员和其他列席会议的同志提出意见。

　　一、2015年和“十二五”工作回顾

　　2015年，面对错综复杂的宏观环境和经济下行压力，在市委的正确领导下，我们坚决贯彻落实中央、自治区稳增长、促改革、调结构、惠民生、防风险各项决策部署，按照“加快建设新城，疏解提升老城，产业融合发展，城乡协调推进，生态文化相融，富裕和谐桂林”的总体要求，坚持国际旅游胜地建设“一本蓝图绘到底”，着力推动有质量的稳定增长、可持续的全面发展，经济运行稳中有进、稳中向好。

　　经济保持平稳增长。着力在扩投资、减负担、促产业、拓市场、优服务等方面，主动作为，精准发力，全面实施稳增长48条工作措施及21项配套政策，主要经济指标平稳较快增长。初步预计，地区生产总值增长8.0%，财政收入增长7.2%，固定资产投资增长19.5%，社会消费品零售总额增长10.1%，城镇居民人均可支配收入增长7.3%，农民人均纯收入增长9.9%，居民消费价格指数涨幅1.9%。服务业提速发展，第三产业增加值增长9.2%，自2003年以来首次快于第二产业增速；工业经济企稳趋优，规模工业增加值增长8.2%，万元工业增加值能耗下降13.7%，每万人口发明专利拥有量增长48.2%；农业生产稳定增长，现代农业发展步伐加快，13个现代特色农业（核心）示范区建设成效明显。

　　项目建设强力推进。狠抓项目规划、用地、融资、管理等关键环节，继续保持项目投资强劲增长势头。全年固定资产投资达1837.3亿元，实施重点项目800项，完成投资764.1亿元，占固定资产投资总量的41.6%。以20个重中之重项目为重点，全面提速重大项目建设。桂林航空公司组建运营，万达广场如期开业，华润万象城开工建设；桂林西客站建成使用，火车南站、北站综合改造整治工程完成；灌阳（永安关）至全州（凤凰）高速公路建成通车，桂林至三江、资源至兴安等高速公路加快推进；国道321桂林至阳朔段改扩建工程市区段基本完成，西二环路重要节点全部打通，福利路二期、万福东路及龙门大桥等项目加快实施，漓江桥改扩建工程、市区道路整修工程、香江饭店立交桥及“两江四湖”二期连通水系等重大项目开工建设；一批县域基础设施、生态环保、社会民生及产业发展方面的项目全面实施，我市发展的综合承载力进一步增强。

　　发展活力持续增强。43项改革任务及19项重点改革专题进展顺利。取消、承接、下放和调整各类行政审批事项195项。在全区率先实行企业注册“全城通、就近办”登记制度，全面落实“三证合一”“一照一码”，有效激活大众创业、万众创新，新登记各类市场主体3.94万户，增长41.2%。金融改革创新成效显著，总额100亿元的国际旅游胜地建设基金成功发行。漓江景区“三统”改革取得阶段性成果，漓江竹筏“四化”专项整治成效明显。积极参与高铁经济带建设，粤桂黔高铁经济带合作试验区（广西园）落户桂林。

　　各项事业亮点纷呈。持续加大民生投入，民生支出占全市一般公共预算支出的75.9%。城镇新增就业6.57万人，农村劳动力新增转移就业7.24万人次，城镇登记失业率3.57%。城市棚户区改造项目开工7989套，开工完成率102.8%，居全区前列。270个贫困村整村推进成效明显，脱贫6万人。筹措资金110.2亿元，完成72项自治区级和市级为民办实事工程。以“五大专项治理”为重点，加大城市环境综合治理力度，大幅提升城管力量，着力改善城管设施，全面整治交通秩序和出租车行业乱象，市容市貌明显改善。国家卫生城市通过复审，全国文明城市创建工作扎实推进。加强生态建设和保护，漓江（城市段）排污综合治理项目年度计划全面完成；PM10、PM2.5年均浓度分别下降18.6%、22.7%，空气质量优良天数同比增加46天。第二批15个新型城镇化示范乡镇“书记工程”、漓江东岸百里生态示范带、传统村落保护利用工程全面推进。加大历史文化保护和挖掘力度，美国飞虎队桂林遗址公园建成开园，逍遥楼重建工程、正阳东巷保护修缮整治工程基本完工，靖江王城“拆围透墙”及城墙修复工程加快实施，文化标识建设全面启动。科技、教育、卫生、计生、体育等社会事业全面进步。扎实推动法治桂林建设，社会保持和谐稳定。过去一年，我市多项工作进入全国全区先进行列，第二次全国改善农村人居环境工作会议、全国旅游厕所工作现场会、自治区生态乡村建设现场会等在我市成功召开。

　　各位代表！随着2015年主要目标任务的完成，我市“十二五”圆满收官，为“十三五”发展奠定了坚实基础。

　　（一）经济综合实力迈上新台阶。去年我市地区生产总值达1943亿元，是2010年的1.76倍，年均增长10.4%；人均生产总值39374元，经济发展总体迈入中等收入阶段。财政收入达209.2亿元，是2010年的1.73倍，年均增长11.6%。累计固定资产投资7059亿元，年均增长23.3%。金融机构存贷款余额分别达2607.1亿元、1580.8亿元，分别是2010年的1.91倍、2.02倍。社会消费品零售总额752亿元，年均增长13.9%。城镇居民人均可支配收入达28768元，年均增长9.9%；农民人均纯收入突破万元大关，达10365元，年均增长13.6%。三次产业结构由2010年的18.4∶44.6∶37.0调整为17.4∶46.4∶36.2。地区生产总值超100亿元的县区由2010年的4个增加到10个，财政收入超10亿元的县区由2010年的1个增加到5个，区域竞相发展格局基本形成。

　　（二）国际旅游胜地建设实现新突破。坚持以桂林国际旅游胜地建设这一国家战略为统领，坚持“一本蓝图绘到底”，着力推动旅游产业转型升级，桂林旅游龙头作用进一步增强。坚持重大项目带动战略，累计实施以旅游为主的服务业重大项目346项，完成投资525.1亿元，项目数量和投资量保持全区领先。进入我市的世界500强、国内500强企业达40家，万达、华润、海航、地中海俱乐部、恒大、悦榕庄、喜来登、海吉星、玉圭园等一批知名企业落户桂林，有力带动了我市旅游整体水平提档升级。4A级以上景区增至33家，居全区第一；星级酒店达68家，床位数比“十一五”末增长21%。特色旅游名县创建工作领跑全区，名镇名村带动的乡村旅游成为桂林旅游新亮点。立体旅游交通网络初步形成。高铁旅游成为桂林旅游增长新一极，高铁游客人数以每年50%以上的速度递增；国际国内航线不断增加和优化，机场旅客年吞吐量保持在600万人次以上；公路通达性显著增强，公路旅客年运输量保持在8000万人次以上。连续举办联合国世界旅游组织/亚太旅游协会旅游趋势与展望国际论坛，中国－东盟博览会旅游展永久落户桂林，每年吸引50多个国家和地区参会代表来桂林交流与合作，成功争取51国公民72小时过境免签、东盟10国旅游团6天入境免签及桂林居民赴台个人游等政策，入境游客人数及旅游收入持续增长，桂林旅游国际化格局加速形成。2015年，旅游接待总人数4470万人次，年均增长12.5%，居全国地级市前列；旅游总收入517.3亿元，是2010年的3.1倍，年均增长24.1%，高于游客人数增幅11.6个百分点，旅游转型升级取得重大突破。

　　坚持以旅游业为龙头，带动服务业加快发展。“十二五”期间，特别是国际旅游胜地建设三年多来，六城区新增大型高端商业项目13个，建筑面积达118万平方米，是我市商贸服务业发展最快的时期。累计新增银行业金融机构5家、保险机构9家，金融服务功能不断增强。动漫产业领跑广西，力港网络公司成为全区首家获得“国家文化产业示范基地”称号的互联网科技企业。商务会展、健康养生养老、现代物流、电子商务等现代服务业快速发展，服务业标准化建设有序推进。引导城市服务业向农村延伸覆盖，农村现代商品流通网络初步形成。2015年，服务业增加值达702.6亿元，年均增长7.6%，服务业税收占全部税收比重达65.6%，比2010年提高14.6个百分点，服务业正成为我市经济发展的新引擎。

　　（三）现代工业发展得到新提升。持续加大投资力度，累计完成工业投资2811.2亿元，是“十一五”时期的3.14倍。福达年产10万吨精密锻件、君泰福智能电网等一批重大工业项目竣工投产，为我市工业发展增添了新动力。累计完成工业园区基础设施建设投资80亿元，新增开发面积18平方公里，新增超百亿元园区3个；2015年，园区工业总产值达1600亿元，占全部工业总产值的64.8%，比2010年提高28.8个百分点。“十二五”末，电子信息、医药及生物制品、先进装备制造、生态食品等四大优势产业完成规模以上工业产值1185亿元，占规模以上工业总产值的50.3%；新能源、新材料等战略性新兴产业完成规模以上工业产值562亿元，占规模以上工业总产值的23.9%，工业结构进一步优化。企业培育提升成效明显，新增产值50亿元以上企业4家；新增上市企业和新三板挂牌企业7家，居全区前列。2015年，全部工业总产值达2469.9亿元，是2010年的1.97倍；工业对GDP增长贡献率达46.2%，工业主导地位更加凸显。

　　（四）“三农”工作取得新成就。各级财政累计投入“三农”资金199.8亿元，是“十一五”时期的2.8倍，也是桂林地市合并以来财政投入“三农”力度最大、农民得实惠最多的五年。现代农业发展取得实质性成效，粮食总产量保持200万吨以上；金桔、砂糖橘、罗汉果、月柿产量全国第一，水果、蔬菜、食用菌产量全区领先；新增国家农产品地理标志登记产品14个。建设“万元增收”示范带6条、种植业标准化生产基地16个、国家级畜禽标准化示范场28家，数量均列全区前茅。休闲农业走在全国前列，“桂林市休闲农业四季游精品线路”获全国休闲农业十大精品线路称号。农业基础设施建设进一步加强，累计完成水利水电投资129.9亿元，是“十一五”时期的2.72倍，桂林市防洪及漓江补水枢纽工程实现川江、小溶江水库下闸蓄水，斧子口水库全面建设；完成中小型病险水库除险加固377座，现有病险水库基本脱险；农业综合机械化率达48.6%，比“十一五”末提高18.7个百分点。新农村建设取得阶段性成果，累计建设普惠制新村2020个，完成新农村试点示范村建设1200个；新增农村标准化公路4109公里、自然村（屯）道路硬化12089公里，建制村通畅率达90%、通公交率达28.7%；累计完成农村危房改造10.1万户，解决30多万农村贫困人口住房问题，建成农村饮水安全工程3313处，惠及160万人；累计建成村级公共服务中心804个，实现村村通广播电视、“村村有书屋”目标。深入实施农民收入倍增计划，城乡居民收入比由3.3∶1缩小至2.8∶1，农民生活水平显著提高。

　　（五）新型城镇化建设实现新跨越。过去五年，是桂林地市合并以来城镇化建设最快的五年，新增城区建成区面积26.2平方公里，常住人口城镇化率达46.7%，比“十一五”末提高7.9个百分点。实施交通优先发展战略，累计完成交通基础设施重大项目建设投资506.6亿元。两江国际机场T2航站楼开工建设。湘桂、贵广高铁相继建成运营，高铁通车里程达420公里，占全区总里程的近四分之一，成为国内首个“一市九站两高铁”的地级市，全面融入粤桂湘黔四省会3小时经济圈。建设高速公路6条，建成2条，通车总里程达450.2公里，实现13个县区通高速公路。二级及以上普通公路里程达1378公里，国省干线公路路网逐步完善，区域性综合交通枢纽地位基本确立。临桂新区建设累计完成投资270.9亿元，实现了“五年上规模”目标。“一院两馆”等一批标志性项目建成使用，行政中心完成搬迁；路网结构基本成型，水、电、气、讯等市政基础设施不断完善，学校、医院、市场、公园等配套项目加快推进，核心区15平方公里基本建成。阳朔、全州、灌阳、龙胜、荔浦、灵川、平乐等县城新区加速建设。老城疏解提升成效显著。以“北通、南畅、东拓、西联”工程为重点的市区路网建设加快推进，累计实施重大项目278项，完成投资283.7亿元，加快了老城新区交通路网的互联互通，外联内畅的城市交通新格局加速形成。累计实施公共服务配套重大项目186项，完成投资198.9亿元。新建及改扩建水厂、供水设施重大项目14项，建成污水管网503.6公里。以福隆园、塔山、福利路片区改造为重点，组织实施19个棚户区改造项目；综合改造城中村89个、无物业管理小区182个，新建改建旅游厕所180座，完善公共自行车、景观照明等公共设施，老城功能品位实现新提升。理顺城市管理体制机制，推行网格化管理新模式；持续加大环境整治力度，市区环境卫生机扫率比“十一五”末提高55.5个百分点，累计清扫面积达1487万平方米；依法拆除“两违”建筑312万平方米；加快“智慧城市”建设，智能交通指挥等系统投入使用，市容市貌、文明秩序得到较大改观。投资82.3亿元，在全区率先实施两批28个各具特色的新型城镇化示范乡镇“书记工程”，辐射带动全市小城镇建设和发展；获广西百镇示范工程16个，数量居全区首位，城乡面貌发生深刻变化。

　　（六）生态文明建设取得新成果。深入贯彻落实习近平总书记关于科学保护漓江的重要指示，严厉打击破坏漓江生态环境的违法违规行为，漓江保护迈入科学化、法制化、规范化的新阶段。桂林喀斯特地貌成功申遗。国家级低碳城市试点工作加快推进，超额完成自治区下达的节能减排目标任务。主要河流水质均达地表水Ⅲ类标准，国控、省控断面水质达标率100%，集中式饮用水源地水质达标率100%，全面完成青狮潭水库网箱养鱼的清理整治；小东江、南溪河治理项目顺利推进。累计投资17.5亿元，新建及改扩建各类污水垃圾处理设施项目54个，市区污水集中处理率达93.5%，城镇生活垃圾无害化处理率超过75%。持续实施“绿满八桂”工程，森林覆盖率达70.9%，恭城、灌阳、阳朔成为国家级生态功能区。“美丽桂林”乡村建设扎实推进，农村人居环境明显改善。累计投入500多亿元，实施重点项目1000项；创建国家级生态乡镇16个、自治区级生态乡镇59个、自治区级生态村151个，数量居全区之首。

　　（七）深化改革开放迈出新步伐。重点领域和关键环节改革取得新突破。积极推进行政审批制度改革，商事制度改革走在全区前列，累计取消、下放和调整权力事项4049项，权力事项精简60.9%。财政体制改革不断深化，市本级单位全部实行预决算公开和三公经费公开。国企改革扎实推进，经营性国有资产集中统一监管体制进一步完善。旅游管理体制改革取得突破，旅游产业用地改革深入推进。集体林权制度主体改革基本完成，农村土地承包经营权确权颁证工作稳步开展。医药卫生体制改革成效显著。文化、教育、科技及社会治理体制改革积极推进。实施“招大引强”战略，累计签订市外境内投资项目1814个，实际到位市外境内资金2789.3亿元，是“十一五”时期的2倍；实际利用外资22亿美元，年均增长10.9%。实施创新驱动战略，累计科研投入110亿元，组织实施科技计划项目4400项，获国家级科技奖4项、自治区级科技奖151项；新增国家农业科技园区、国家级文化与科技融合示范基地、国家级大学科技园等国家级科技创新平台6家；高新技术企业、创新型企业达到116家，每万人口发明专利拥有量达4.6件，居全区前列，科技创新能力持续增强。

　　（八）民生和社会事业得到新改善。民生领域累计投入1060.7亿元，年均增长16.6%，办成了一大批关系群众切身利益的实事好事。累计城镇新增就业40.5万人、农村劳动力新增转移就业45.8万人次。学前教育三年毛入园率、九年义务教育巩固率、高中阶段教育毛入学率分别达83.9%、97.07%、90.89%，全面实现城乡免费义务教育，5个县实现高中阶段免费教育，职教攻坚实现新突破，桂林航专、旅专升本。新农合、城乡居民养老保险参保率分别达99.54%、97.9%，城镇基本医疗保险覆盖率达92%。开工建设城镇保障性住房6.98万套，是“十一五”时期的3.81倍。累计发放低保金23.9亿元。累计投入扶贫资金57亿元，是“十一五”时期的3倍，减少贫困人口34万人。深入实施“寻找桂林文化的力量，挖掘桂林文化的价值”工程，对桂林历史文化进行系统挖掘和整理，靖江王府及王陵、甑皮岩两个国家考古遗址公园建设加快推进，百姓大舞台、漓江之声等公共文化品牌影响力进一步增强；本土原创动漫剧《可可小爱》在央视播出，开辟出一条以现代动漫为载体、以弘扬社会主义核心价值观为重点的文化发展新路子。县、乡（镇）、村三级基层医疗卫生服务体系逐步健全，社区卫生服务机构实现城区全覆盖。体育事业蓬勃发展，全民健身活动扎实推进。安全生产形势持续稳定，食品药品监管得到加强。“六五”普法圆满完成，应急处置能力有效提升，社会治安综合治理成效显著，“平安桂林”建设再上新台阶，人民群众安全感明显增强。

　　五年来，国防建设及双拥工作得到加强，民族、宗教、外事、侨务、对台工作、人事、审计、监察、统计、物价、编制、接待、机关事务、档案、供销、保密、口岸、海关、海事、气象、水文、测绘、地方志、检验检疫、防震减灾、决策咨询、社会科学、残疾人等事业取得新进步，中直、区直驻桂单位取得新成绩，工会、共青团、妇联等群团组织在经济社会发展中发挥了重要作用。

　　五年来，我们全面推进依法行政，建设法治政府、廉洁政府。认真执行人大及其常委会的决定、决议，自觉接受人大、政协和社会监督，人大代表建议、政协提案办复率达100%。深入开展党的群众路线教育实践活动和“三严三实”专题教育，严格贯彻落实中央“八项规定”和国务院“约法三章”，廉政建设和反腐败斗争取得新成效，党群干群关系更加密切。

　　各位代表！五年来，我们共同生活的城市发生了深刻的变化，桂林国际旅游胜地建设取得了重大突破。这些成绩的取得，凝聚着全市人民的辛勤劳动，体现着社会各界的贡献付出。在此，我代表市人民政府，向全市各族人民，向人大代表、政协委员，向各民主党派、工商联、无党派人士、各人民团体和各界人士，向驻桂部队和武警官兵，向所有关心支持桂林发展的海内外朋友，致以崇高的敬意和衷心的感谢!

　　与此同时，我们也清醒地认识到存在的问题和面临的挑战：经济总量小，人均水平低，产业不大不强，创新能力不够突出，发展动力不足，实现经济持续稳定增长任务艰巨；城乡发展不平衡，农村基础设施和公共服务设施建设滞后，推进城乡统筹协调发展既有较好基础、又面临艰巨任务；资源环境约束趋紧，生态环境保护任务繁重；城市管理有待进一步提升，市民文明素质和社会文明程度有待提高；制约发展的体制机制障碍亟待破解，干部队伍思想作风和能力水平有待加强等等。面对前进中的困难和问题，我们必须增强危机意识和赶超意识，必须不断改进工作作风，提升政府工作水平，用新理念、新办法推动各项事业全面发展。

　　二、“十三五”时期的奋斗目标和主要任务

　　“十三五”时期是我市全面建成小康社会的决胜期，是基本建成桂林国际旅游胜地的攻坚期，也是我市由中等收入向中高收入跨越、工业化中期向中后期发展的重要阶段，发展机遇与挑战并存。我们要牢牢把握桂林国际旅游胜地建设正式纳入“双核驱动、三区统筹”战略布局、上升为自治区核心战略的重大机遇，进一步增强发展信心和担当意识，有效应对各种风险挑战，努力走出新常态下具有桂林特点的发展之路。

　　根据《中共桂林市委员会关于制定国民经济和社会发展第十三个五年规划的建议》（以下简称《“十三五”规划建议》），市人民政府在广泛征求人大代表和政协委员、各县区、各部门、各民主党派、专家学者以及社会各界意见的基础上，制定了《桂林市国民经济和社会发展第十三个五年规划纲要（草案）》［以下简称“《纲要（草案）》”］。整个起草过程充分体现了科学决策和民主决策的要求，是集体智慧的结晶。《纲要（草案）》共分3大板块14章48节。第一章构成第一板块，分析“十三五”发展环境，明确指导思想、发展目标和发展动力。第二章至第十三章构成第二板块，就我市经济社会发展各领域进行全面部署。第十四章为第三板块，提出保障措施。

　　（一）“十三五”经济社会发展总体要求和目标

　　我市“十三五”时期发展的总体要求是：高举中国特色社会主义伟大旗帜，全面贯彻党的十八大和十八届三中、四中、五中全会精神，以马克思列宁主义、毛泽东思想、邓小平理论、“三个代表”重要思想、科学发展观为指导，深入学习贯彻习近平总书记系列重要讲话精神，紧紧围绕“四个全面”战略布局，牢固树立创新、协调、绿色、开放、共享发展理念，主动融入自治区“双核驱动、三区统筹”格局，坚持桂林国际旅游胜地建设“一本蓝图绘到底”，按照“加快建设新城、疏解提升老城、产业融合发展、城乡协调推进、生态文化相融、富裕和谐桂林”的总体要求，加快推进新型工业化、城镇化、信息化和农业现代化，更加注重发展质量和效益，更加注重改革创新，更加注重保障改善民生，全面推进经济建设、政治建设、文化建设、社会建设、生态文明建设，确保如期全面建成小康社会和基本建成桂林国际旅游胜地，为谱写中华民族伟大复兴中国梦的桂林篇章奠定更加坚实的基础。

　　与全国全区同步全面建成小康社会、基本建成桂林国际旅游胜地，这是市委《“十三五”规划建议》提出的、必须奋力实现的“两个建成”宏伟目标，与自治区提出的“两个建成”目标既一脉相承，又具有鲜明的桂林特色，符合桂林实际和发展趋势。与全国全区同步全面建成小康社会，包括经济发展、创新驱动、民生福祉、生态文明四大类33项具体指标，其中最关键的就是经济增速要高于全国、全区平均水平，确保到2020年实现地区生产总值和居民人均可支配收入比2010年翻一番的目标，年均增长分别达到8%、10.4%以上。提出这一目标，主要基于以下几个方面的考虑：一是实现“两个建成”目标，必须有一定的发展速度作保证。目前，我市人均生产总值只有全国的80%，如果增速不比全国、全区高一些，不仅财政、就业、民生、公共服务、扶贫等方面将受影响，延缓全面建成小康社会的进程，还会进一步拉大与全国的发展差距。二是实现上述目标，我市有条件、有基础。根据测算，“十三五”期间，我市地区生产总值潜在增长率为8.5%左右。从供给支撑条件看，农业增加值年均增长3%，工业增加值年均增长9%，服务业增加值年均增长11%；从需要支撑条件看，固定资产投资年均增长13%，社会消费品零售总额年均增长10%，这些目标通过努力是有把握实现的。从居民收入水平来看，随着“十三五”收入倍增计划实施、消费升级加快、脱贫攻坚任务完成，我市居民人均可支配收入年均增长10.4%也是可以实现的。特别是近几年我市打下了较好的项目基础，“十三五”时期，一批大项目、好项目将相继建成，发展动力将进一步增强。三是既要稳定增长，又要加快结构调整。“十三五”是经济发展在新常态下运行的重要时期，按照创新、协调、绿色、开放、共享的发展理念，对经济增长要求更高，要改变过去粗放型增长方式，更加注重创新驱动，更加注重结构调整，更加注重发展质量和效益，实现更有质量、更高效益和更可持续的发展。综合分析，地区生产总值8%、城乡居民人均可支配收入10.4%的增长目标以及其他发展目标要求，总体上是科学合理、切实可行的。

　　各位代表，实现上述目标，到2020年，我市将与全国全区同步全面建成小康社会，基本建成桂林国际旅游胜地，成为世界一流的旅游目的地、全国生态文明建设示范区、全国旅游创新发展先行区、区域性文化旅游中心和国际交流的重要平台、“一带一路”有机衔接的综合交通节点，经济发展将总体迈进中高收入阶段，新型工业化、城镇化、信息化、农业现代化和绿色化水平进一步提升，产业向中高端发展，现行标准下农村贫困人口全部脱贫，人民生活将更加富裕安康，与全国平均发展水平差距进一步缩小，生态环境保持全国一流，美丽桂林将焕发出更加灿烂的光彩。

　　（二）“十三五”重点任务

　　坚持创新发展、科学发展、改革开放、法治保障、民生为本原则，针对我市经济社会发展的关键瓶颈和薄弱环节，突出抓好七大重点任务的落实。

　　实施产业做强升级攻坚战，构建现代产业新体系。以特色主导产业为支撑，改造提升传统产业，大力发展高新技术产业、旅游业、现代服务业和现代农业，推进产业集聚发展，打造桂林产业升级版。

　　——推动工业迈向中高端。全面落实“中国制造2025”，推进“两化”深度融合，推动桂林制造向“桂林智造”、桂林创造迈进。创新园区管理体制机制，推动企业向园区集聚，打造高新技术开发区、桂林市经济技术开发区“两翼齐飞”，各县工业集中区“多点支撑”的工业园区发展新格局。加快提升电子信息、医药及生物制品、先进装备制造、生态食品等优势产业主导地位，积极培育新一代信息技术、节能环保、新材料、大健康等新兴产业，改造提升包装与竹木加工、建材、电力、橡胶化工、冶金等传统产业。加强质量品牌建设，实施“互联网+制造”和“互联网+中小微企业”行动计划，强化工业基础能力。到2020年，实现全部工业增加值1300亿元以上。

　　——提升桂林旅游核心竞争力。加强旅游设施和服务体系建设，构建人性化的服务体系。实施“品质旅游”战略，构建“一核三极三带”旅游空间布局，打造多样化旅游精品。推动产业融合，提升产业素质，壮大旅游企业整体实力。构筑旅游开放合作新平台。深化智慧旅游建设，培育“旅游+”新业态。到2020年，实现旅游总收入1000亿元以上。

　　——提升服务业发展质量和效益。创新“互联网+服务业”新业态，规划建设一批现代服务业集聚区，促进健康养生、商贸、运动休闲等生活性服务业提质升级，推进电子商务、信息、物流、金融、商务会展等生产性服务业做大做强，以标准化引领服务业发展。到2020年，服务业增加值占地区生产总值比重达到45%以上。

　　——加速推进农业现代化。加快转变农业发展方式，着力构建现代农业产业、生产和经营体系，加快推进适度规模经营。夯实农业基础，稳定粮食产量，大力发展循环农业、绿色有机农业、富硒农业，培育休闲农业、生态农业和都市农业等新业态，做强优势特色农业。创建一批现代特色农业（核心）示范区，推广“互联网+现代农业”新模式，促进农业“接二连三”融合发展，实现由农业大市向农业强市的跨越。到2020年，实现农业增加值400亿元以上。

　　实施基础设施建设攻坚战，构建现代基础设施支撑体系。抢抓国家实施“一带一路”和“宽带中国”战略机遇，加强交通、能源、水利、信息和城市基础设施建设，提升城市可持续发展的支撑能力。

　　——打造“一带一路”有机衔接的综合交通节点城市。加大交通基础设施建设力度，强化对外衔接能力，提高综合运输服务水平。完成两江国际机场扩建工程，规划建设一批通用航空机场；加快推进湘桂铁路既有线电气化改造，建设桂林北动车运用所、桂林西货运中心；推进高速公路建设，实现县县通高速。优化市域交通网络，推进连接产业园区、旅游景区等重要节点的道路建设。加快推进桂江（漓江）航道工程建设。

　　——加强能源保障。调整能源结构，加强城市配电网建设，不断提升地方电力保障能力。开发、推广应用清洁能源和可再生能源，加快以风电为代表的电力和配套送出工程建设，建成一批风电场。发展分布式能源。继续推进天然气推广利用，实现县县通管道燃气。

　　——加强水利基础设施建设。完成主要大中型病险水闸除险加固。完善小型农田水利建设，推进大中型灌区续建配套和节水改造。加快中小河流治理，完善城镇防洪设施。

　　——加快建设智慧桂林。实施大数据战略，完善信息化基础设施，建设城市公共信息平台。建设新一代移动通信网、下一代互联网和数字广播电视网，实现“三网融合”。提升信息化技术应用水平；完善农产品电子交易平台。加快建设信息惠民国家试点城市和中国－东盟信息港桂林数据备份中心。

　　——提高中心城市承载能力。基本建成临桂新区，基本完成老城疏解提升。完善城市基础设施，增强城市公共服务能力和水平。加强市政公用设施建设，完成一批重要交通节点和道路、桥梁、综合管廊等工程建设；加快城市大公交系统建设，创建公交都市；建设航空、铁路、公路与城市公交之间的衔接工程，实现“零距离换乘”，创建全国综合运输服务示范城市。推进城市环境综合整治。统筹规划城镇学校、医院、文化、体育等公共服务设施建设。

　　实施脱贫攻坚战，推进精准扶贫精准脱贫。这是我市“十三五”时期最大的政治责任、最大的民生工程、最大的发展机遇，必须全力推动，确保到2019年龙胜、资源和灌阳3个贫困县（片区县）全部“摘帽”，到2020年全市28.7万农村贫困人口和返贫人口实现脱贫，499个贫困村出列。

　　——精准实施“七个一批”脱贫攻坚。扶持生产发展一批、转移就业扶持一批、移民搬迁安置一批、生态补偿脱贫一批、教育扶智帮助一批、医疗救助解困一批、低保政策兜底一批。

　　——全面推进脱贫攻坚“十大行动”。特色农业富民行动、特色旅游扶贫行动、基础设施建设行动、扶贫移民搬迁行动、农村电商扶贫和农民工培训创业行动、金融扶贫行动、贫困户资产收益扶贫行动、科技文化扶贫行动、社会扶贫行动、农村“三留守”和残疾人关爱服务行动。

　　——完善脱贫工作机制。建立扶贫大数据管理平台。完善扶贫财政投入稳定增长机制，加大对贫困村财政支持力度。强化脱贫工作责任考核。

　　深化改革开放，增强经济社会发展新动力。以扩大开放深化改革发展，以深化改革促进扩大开放，建立完善创新创业体制机制，为经济发展注入新动力。

　　——全面深化改革。加快推进行政体制、财税体制、国有企业、投融资体制、旅游管理体制、土地制度及科技、教育、文化、卫生、社会保障等重点领域和关键环节改革，着力破除转型发展的深层次问题和结构性矛盾，形成有利于科学发展的新机制。

　　——扩大开放合作。实施更加主动的开放战略，加强区域合作和内外联动，扩大国际交往。实施外贸振兴计划，提升外贸竞争力。加大招商引资力度，创新外资利用方式，扩大利用外资规模。积极参与广西构建面向东盟的国际大通道、打造西南中南地区开放发展新的战略支点、“一带一路”战略有机衔接的重要门户建设，积极融入珠江－西江经济带，按照“五年见成效，十年基本建成”目标要求，加强粤桂黔高铁经济带合作试验区（广西园）建设，打造多区域合作发展的新高地。

　　——推动创新创业。实施创新驱动发展战略，构建有利于大众创业、万众创新的政策环境，搭建创新创业平台，强化创新人才队伍建设，实施产业高级人才引进工程，培育创新型企业，创建国家级自主创新示范区。到2020年，自治区级以上企业技术中心达到80家，培育高新技术企业150家。

　　推进协调发展，开创城乡统筹新局面。坚持集约发展，改善城镇生态环境，提高城镇发展持续性、宜居性。到2020年，常住人口城镇化率达到55%。

　　——促进城镇协调发展。推进中心城市组团式发展，打造功能合理、特色鲜明、优势互补、差别发展的中心城区六大组团。以湘桂走廊和贵广高铁沿线城镇轴为中心，实施中小城市培育工程，发展中小城市和城镇，构建产业分工合理、城市功能互补、城乡协调发展的桂北城镇群，打造桂林都市圈。

　　——推动产城融合发展。推进产业发展与城镇建设互动、产业升级与城镇转型协同、产业集群与城镇集群融合，促进产业和人口向城镇集聚。

　　——提升县域经济实力。发挥各县资源优势，积极融入区域都市经济圈，发展特色产业经济，加速产业集聚，做大县域主导产业，着力打造独具特色的新型产业基地。

　　——改善农村人居环境。以环境综合整治和基础设施建设为重点，全面改善农村生产和人居环境。保护和修复村屯原始风貌和自然景观、田园景观。推进绿色村屯建设，实施连村通屯道路工程，开展污水垃圾专项治理和土壤环境整治，推进农村饮水安全巩固提升工程。加快发展中心村，整治“空心村”，扎实推进新农村建设。

　　实施绿色发展，打造生态经济新高地。全面实施“生态立市”战略，加强生态建设和环境保护，着力推进国家低碳城市试点建设，打造国家生态文明示范市。

　　——加强生态建设。强化重点生态功能区和自然保护区管护，实施重大生态保护修复工程，抓好重要生态系统和生态脆弱地区保护修复。加强漓江保护，2020年前，彻底解决漓江市区段面源污染问题；完善漓江管理体制，建立健全漓江流域生态补偿机制。

　　——加强环境保护。建设水生态文明试点城市。加强城镇污水处理和城乡饮用水源地保护。强化工业废气、机动车尾气和城市扬尘源等污染治理。加大对生活垃圾、医疗废弃物、工业固体废物、城乡建筑垃圾无害化处理。推动绿色建筑发展，创建绿色交通城市，推进生活方式绿色化。

　　——大力发展生态经济。加快园区生态化改造，建设循环产业园区。对传统工业进行绿色化改造，构建工业生态产业链；推进全国循环农业示范市建设；以低碳化为导向，发展现代服务业，构建生态经济体系，建成广西生态经济发展先行区。

　　坚持共享发展，全面实现小康社会新期盼。加强民生和社会事业建设，健全基本公共服务体系，提高基本公共服务均等化水平，提升城乡居民福祉，实现全市人民群众共同迈入全面小康社会。

　　——促进教育协调发展。实施学前教育普及工程、义务教育均衡发展工程、普通高中特色发展工程、职业教育突破发展工程。新建桂林职业技术学院。支持驻桂高校建设，完成桂林师专升本工作。建设面向东盟的人才教育培训基地。

　　——彰显桂林文化新魅力。实施“寻找桂林文化的力量，挖掘桂林文化的价值”工程。加大历史文化遗产保护力度；完善公共文化服务网络，办好群众文化活动；实施文化精品工程，丰富优秀公共文化产品供给，繁荣文化事业。以文化创意、出版发行、演艺娱乐等为重点，发展文化产业。加强精神文明建设，弘扬社会主义核心价值观。

　　——提高城乡居民健康水平。完善公共卫生服务体系，加强综合性医院和专科医院建设；继续推进县（区）、乡镇（社区）、农村基层医疗卫生机构建设；开展爱国卫生运动；加大对传染病及慢性病防控力度；加强全科医生、儿科医生培养和乡村医生队伍建设；推进中医药事业发展。加强食品药品安全监督体系和监管监测能力建设，推进医疗器械检测中心建设。开展全民健身活动，完善城市社区、乡镇、农村体育健身场所和设施，发挥体育场馆和设施作用。

　　——完善社会保障。坚持就业优先战略，健全市、县(区)、乡镇（街道）三级公共就业服务体系。完善工资收入稳定增长机制，提高城乡居民收入。建立城乡一体的社会保障体系，实施全民参保计划，基本实现法定人员社会保险全覆盖。大力发展社会福利事业，加强社会救助能力建设。全面落实优抚安置政策；做好拥军优属工作。

　　——提升社会治理水平。创新社会治理模式，完善社会治理体制机制，深化公共安全建设。加强法治建设，坚持依法行政，到2020年，基本建成职能科学、权责法定、执法严明、公开公正、廉洁高效、守法诚信的法治政府。

　　各位代表！“十三五”规划的宏伟蓝图凝聚着社会各界的智慧，承载着全市人民的梦想。我们要俯身耕耘，顽强拼搏，朝着“两个建成”的目标阔步前进，携手开创更加美好的未来！

　　三、2016年的主要目标与重点工作

　　今年是“十三五”的开局之年。我市经济社会发展主要预期目标是：地区生产总值增长8%－8.5%；财政收入增长6.5%－7%；固定资产投资增长15%－16%；社会消费品零售总额增长11%－12%；城镇居民人均可支配收入增长9%，农民人均纯收入增长10%；居民消费价格涨幅控制在3%左右；城镇登记失业率控制在4.5%以内；人口自然增长率控制在11‰以内；主要污染物排放量控制在自治区下达目标内。为完成上述目标，我们要积极进取，统筹兼顾，奋发有为，以超常的勇气，采取超常的措施，重点抓好以下工作，为实现“十三五”宏伟目标开好局、起好步。

　　(一)加快推进产业做强升级

　　以高新技术产业为龙头加快发展现代工业。优化产业空间布局，推进产城融合发展。全面启动桂林市经济技术开发区建设，理顺管理体制，改革运营机制和投融资模式，统筹各方利益关系，构建以苏桥经济开发区、秧塘科技园和宝山工业园三大核心园区为主体的“一区三园”新格局，确保年内初见成效；积极拓展高新区产业发展空间，加快光通信产业园、花江生态科技园等专业园区建设，做大铁山园、信息产业园、英才园规模；加快推进雁山低碳经济产业园等城区及县域特色产业园发展。加快产业做强升级。以四大优势产业和战略性新兴产业为重点，继续推进智能制造试点示范专项行动；着力推进“互联网+制造”模式；继续推动中小企业实施网上营销工程，力争新增50家工业企业开展网上营销；建成国家新型工业化电子信息产业示范基地；加快推动新能源汽车产业发展，力争四大优势产业规模工业产值达到1300亿元。启动实施新一轮技术改造升级工程，全面提升产品技术、工艺装备、能效环保等水平。实施“招大引强”战略，建立市县（区）、园区、企业互联互动机制，瞄准行业龙头企业，开展点对点精准招商，围绕延长产业链、做大产业集群目标，引进一批产业关联度高、集群效果好、利税增收能力强的项目。组织实施“百亿”工业项目培育工程，力争工业投资增长15%。突出问题导向，加大对企业扶持服务力度，积极支持企业上市，多渠道解决企业筹资难、融资贵等问题，落实好自治区降本增效专项行动方案；重新梳理扶持政策，重点支持发展后劲足、就业和税收贡献大、生态环保的企业做大做强。通过努力，实现规模以上工业增加值增长9%－9.5%，进一步增强工业的核心竞争力。

　　以旅游业为龙头加快发展现代服务业。围绕胜地建设第二阶段目标任务，大力实施旅游发展“三大工程”。实施“项目带动工程”。开工建设万达文化旅游城等重大项目，加快推进华润万象城、阳朔瑞盛旅游休闲世界等项目建设，引进培育一批示范性、创新性强的新业态项目，持续推动旅游国际化、高端化、精品化、特色化发展。实施“产业提升工程”。顺应市场需求，重点发展生态休闲旅游、低空旅游、体验旅游、体育旅游、自驾车营地旅游、摄影旅游等新业态，培育个性化高端观光线路。扎实开展广西特色旅游名县名镇名村创建工作；恢复、重建、改造一批特色街区，打造以王城王府文化、正阳东西巷名人文化等为代表的历史文化产业聚集区；推进乡村旅游提档升级，挖掘、保护、开发一批古村落，打造旅游新亮点。实施“环境优化工程”。深化漓江管理体制、经营体制、分配体制改革，全面启动漓江市区段环境综合保护工程，大力整治旅游市场秩序，整合漓江游船经营企业，完成漓江竹筏“四化”专项整治和精华段游船改造。加强旅游公共服务基础设施建设，重点推进桂林北站游客中心等项目，完善旅游换乘接驳、信息咨询服务，加强旅游厕所建设和管理，提升旅游服务档次和质量。深化旅游区域合作，推动建立粤桂湘黔四省区旅游合作联盟。办好第十届联合国世界旅游组织/亚太旅游协会旅游趋势与展望论坛和第二届中国－东盟博览会旅游展等活动。启动20个市级服务业集聚区建设，推进海吉星食尚港、桂林电商谷等重点项目。加快推进电子商务进农村、进社区，实现电子商务全覆盖。积极开展百店大促销等活动，多措并举促进消费增长。加快发展健康养生养老、文化创意等现代服务业。大力发展服务业限上企业，扎实推进“个转企、小上限”工作。力争实现服务业增加值增长10.5%－11%、旅游总收入增长20%，推进服务业发展提速、比重提高、水平提升。

　　以特色效益农业为龙头加快发展现代农业。强化粮食安全责任落实，确保粮食年产量稳定在200万吨以上。实施现代特色农业“7+3”提升行动，提升粮食、水果、蔬菜、食用菌、茶叶、肉牛肉羊、生猪七大种养产业，着重发展富硒农业、有机循环农业、休闲农业三个新兴产业，突出发展西部山区高山农业。创新生态养殖桂林模式，推进养殖业转型升级，打造标准化规模养殖及健康养殖、草地生态畜牧业、特色养殖三大示范带。巩固提升木材加工等传统优势产业，抓好林下经济示范项目建设。继续做好休闲农业线路开发，促进乡村旅游与精品农业、特色农业相融合，发展星级农家乐、家庭农场、农事体验等休闲农业产品，打造10个以上赏花基地，举办10个以上花节活动。市本级投入1650万元扶持现代特色农业（核心）示范区创建工作，基本建成自治区级、市级示范区10个。全面开展农村土地承包经营权确权颁证登记工作，引导和规范土地有序流转；市本级投入1000万元用于培育各类新型农业经营主体和服务主体，每个县扶持培育5－10个农民合作社示范社。加快农业标准化进程，完善农产品质量体系，抓好“三品一标”基地建设。力争农业增加值增长3%、农民人均纯收入达到11400元，继续保持农民收入、农业产业结构调整、休闲农业发展在全区的领先地位。

　　（二）强力实施项目带动战略

　　全力推进重点项目建设。牢固树立抓项目就是抓发展、抓财源、抓后劲的理念，以自治区层面统筹推进的重大项目及市领导包抓的12大重中之重项目为抓手，努力扩大有效投资。实施第一批重大项目787个，年度计划投资747.6亿元。其中实施重大基础设施项目277个，年度投资276.4亿元；实施重大产业项目411个，年度投资423.8亿元。继续加大社会民生、生态环保领域投资力度。重点推进两江国际机场T2航站楼建设；开工建设桂林北动车运用所、桂林西货运中心；加快桂林至三江、资源至兴安、阳朔至鹿寨等高速公路建设步伐，开工建设荔浦至玉林高速公路，力争开工建设灌阳至平乐高速公路；全力推进龙脊梯田大循环旅游公路等5条国省干线公路的升级改造；加快推进磨盘山客运港改扩建工程；加快斧子口水库建设，基本建成桂林市防洪及漓江补水枢纽工程，继续实施中小河流治理工程；加快推进临桂新区华能分布式能源工程等项目建设。通过这些项目的实施，使我市交通、水利、能源等基础设施再上一个新台阶。

　　优化完善项目推进机制。巩固行政审批制度改革成效，深化“马上办”“五个一”工作机制，优化投资项目审批流程，推进审批标准化工作，加快项目审批进度，进一步完善项目滚动管理和退出机制。强化市领导跟踪联系项目的统筹指挥功能，强化重大项目办公室统筹协调督查的职能，强化项目指挥部对重大项目推进落实的直接责任，及时协调解决重大项目推进过程中存在的突出问题，确保项目早开工、快建设、早投产。

　　强化重大项目要素保障。多途径破解环境制约和土地制约，大力清理闲置土地，优先保障重大项目土地供给。认真做好项目规划，切实加强项目包装策划。抓好中央投资项目资金的争取与管理工作，清理项目沉淀的财政资金，盘活财政存量资金。创新融资产品，拓宽融资渠道。加大资本市场利用力度，扩大直接融资比重；加强市、县（区）资产的整合与管理，完善政银企对接机制，提高间接融资比重。建立健全市县两级融资偿还机制，强化融资风险管控，维护良好的金融生态环境。

　　（三）加快提升城乡品质

　　完善城市服务功能。进一步疏解提升老城。继续实施“北通、南畅、东拓、西联”工程。基本完成国道321桂林至阳朔段改扩建项目，全面完成西二环路延长线、福利路、龙门大桥、万福东路建设，形成南北道路通畅、东西快环连通的老城新区路网新格局。完成漓江桥改扩建、香江饭店立交桥建设等工程，加快将军桥改造；完成东二环路等市区45条主要道路整修工程，启动城市“断头路”连通工程；加快桂林汽车客运总站搬迁步伐。通过一系列综合措施，加快破解老城区交通瓶颈。继续坚持以项目建设带动老城改造的思路，实现改造一个片区、新增一个业态、惠及一方市民、提升一片形象的目标。加大以福隆园、塔山片区为重点的漓江东岸棚户区和城中村改造力度，力争年内初见成效；继续实施面上棚户区改造，开工建设保障性住房22243套，其中建成6000套，分配入住5000套，实现老城提升新突破。围绕单位入驻、市民入居目标，加快完善临桂新区城市功能。强化行政中心带动效应，加快市直机关搬迁步伐，推进市民广场、山水公园、旅游综合医院、桂林中学临桂校区、桂林师专临桂校区、集贸市场等项目建设，力争实现金融大厦、建设大厦、投资发展商务大厦、桂林日报社传媒中心等项目建成投入使用，增加上下班时段公交车发车密度，不断完善水、电、路、气、讯等市政设施，提升新区综合配套功能。

　　推进城市综合治理。加快出台市民文明公约，健全城市管理法规。完善城市管理体制，明确城区政府的主体地位，保障管理资源进一步向城区、向一线倾斜。强化抽查通报、检查问责和公开曝光制度，确保城区的主体责任与市直相关部门的统筹监管责任落实到位。启动实施亮化改造提升工程、特色旅游街区城市风貌改造工程。升级环卫设施，进一步提高机扫率。扎实推进“两违”整治工作。继续抓好“智慧城市”建设。加快公交都市、智能交通、绿色交通发展，扎实开展交通运输综合行政执法改革试点工作和出租车专项整治行动。加强城市环境综合整治，继续抓好无物业小区及集贸市场改造，开展乱摆摊点、乱设广告、乱倒垃圾“三乱”综合治理行动。强化城市规划的刚性约束力，做好城市功能布局，完善城市标线、标识、标牌，提高城市精细化管理水平。

　　加快特色城镇和新农村建设。实施大县城战略，加快县城老城提升改造和新区建设步伐，推进广西乡土特色建设示范县和自治区级新型城镇化示范县创建工作。推动灵川八里街片区与市区一体化规划管理。完成第二批15个新型城镇化示范乡镇“书记工程”，扩大“百镇示范工程”建设覆盖面。启动实施农村饮水安全巩固提升工程。加快推进“通畅”“连通”工程，新增通自然村（屯）公路200公里以上，实现有条件的县（区）建制村100%通沥青（水泥）路，20户以上自然村（屯）通路率达65%；推进17个传统村落保护发展示范村建设。

　　（四）加强生态环境建设

　　狠抓环境治理。坚持以改善环境质量为核心，加强环保能力建设，实行最严格的环境保护制度。严格落实环境保护目标责任制。分解落实自治区下达的“十三五”节能降碳和污染减排目标任务，全面加强生态环境建设，万元生产总值能耗及二氧化碳排放量、主要污染物排放量控制在自治区下达目标内。实施水污染防治行动计划，推进全国水生态文明城市建设试点工作。加快城区污水处理厂提标改造，继续推进乡镇污水处理厂建设；实施漓江环境综合治理与生态保护工程，重点推进53个漓江城市段截污工程项目；开展市区黑臭水体整治，力争实现市区污水直排口全部截污，确保市区生活污水集中处理率达95%以上。推进农村冲水厕所、无害化厕所改造建设。推广生态养殖等清洁生产技术，加强农业面源和养殖业污染治理。加强建筑工地施工监管。继续实施防治大气污染“五大专项行动”，将砖厂、采石场、燃煤小锅炉整治扩展到全市，推广应用新能源汽车，完成自治区下达的黄标车和老旧车淘汰任务；加强大气监测和重污染天气预警预报力度，对市区改善空气质量实施“PM10+PM2.5”联合控制，力争PM10年均浓度降至每立方米67微克以下。加强重金属污染防治。严格危险化学品环境管理。加强企业污染物排放监控，依法依规治理污染，严厉处罚偷排、超排行为。重点推进生活垃圾“三化”综合处理、特殊废弃物处理等工程建设，城市生活垃圾无害化处理率保持100%。

　　加强生态建设。深入开展“美丽桂林?生态乡村”活动，启动建设“宜居乡村”，实施农村垃圾处理两年攻坚行动，力争90%以上的村庄垃圾实现有效处理，建设自治区级绿化示范村屯567个。市本级安排2000万元，支持建设200个生态乡村示范村。继续强力推进漓江东岸百里生态示范带，每个县打造1－2条生态示范带。加快农村环境连片整治，进一步改善农村人居环境，打造美丽乡村升级版。深入实施石漠化治理、新一轮退耕还林等重点工程，全面停止国有林场天然林商业采伐，完成植树造林13.2万亩，力争森林覆盖率达70.93%。

　　（五）积极推动改革创新

　　进一步深化重点领域改革。落实好自治区相关改革部署，按照优化存量、引导增量、有效减量的要求，加快去产能、去库存、去杠杆、降成本、补短板，推进结构性改革，努力增加有效供给。抓紧制定实施企业重组、“僵尸企业”处置方案，加大力度淘汰落后产能。按照自治区要求抓好住房制度改革，着力解决部分商品房库存问题，稳定房地产市场。通过降费、让利等措施，降低企业生产经营成本。继续深化行政审批制度改革。抓好园区管理体制机制改革，深化国资国企改革。推进投融资体制改革，整合创新投融资平台，完善融资服务和政银企合作机制，保障重点园区、重点产业、重点项目融资。积极推进桂林银行等企业上市。推进旅游综合改革，加快旅游企业集团化发展和旅游资源集约化开发。加快理顺市与城区责、权、利关系，构建事权与财权相匹配的市与城区管理体制。加快农村土地制度改革。抓好生态领域改革。推进社保、文化、教育、科技、卫生、计生等民生领域改革。加快户籍制度、网络化管理等社会治理体制改革，促进社会和谐稳定。

　　进一步强化开放带动和创新驱动。研究制定桂林实施全区“3+1”国家战略全覆盖的工作方案，综合推进粤桂黔高铁经济带合作试验区（广西园）、湘桂合作示范区建设。继续实施加工贸易倍增计划，扩大医药、机电和绿色农产品出口。深入实施人才强市战略，加快重大人才工程建设，培育和引进一批高层次人才和创新团队。大力推进桂林自治区级自主创新示范区建设，重点加强桂林国家农业科技园区、桂林文化和科技融合示范基地、大学科技园等创新平台建设。充分发挥驻桂高校和科研院所的资源优势，加强与知名高校合作，促进协同创新和成果转化。加强知识产权创造、运用与保护，力争全年申请发明专利4800件，每万人口发明专利拥有量达5件。积极创建全国质量强市示范市。

　　（六）打好脱贫攻坚战

　　全面完成精准识别建档立卡，建立大数据管理平台，组织实施脱贫攻坚“七个一批”“十大行动”，确保减少农村贫困人口7.6万人，实现100个贫困村脱贫。扎实开展“村企共建”和“千企扶千村”活动，认真实施“一村一策”“一户一法”。统筹市县两级政府各类涉农资金，确保扶贫专项资金足额安排。完善贫困乡村基础设施，因地制宜解决通路、通水、通电、通网络等制约脱贫的突出问题。加大富民产业培育力度，做大做强畜禽养殖等特色种养业；实施旅游扶贫工程，做精做优“农家乐”“生态民居旅馆”等品牌，着力打造一批旅游扶贫示范点。完善脱贫工作责任制，强化责任考核，确保精准脱贫攻坚工程取得实效。

　　（七）着力增进人民福祉

　　坚持发展为民，多办顺应民意、化解民忧、为民谋利的实事好事，让群众拥有更多获得感。

　　大力推进“双创”工作。抓好大众创业、万众创新，扎实推进高新区“众创空间”和雁山区大学生创业基地建设，做好荔浦、兴安农民工创业基地建设试点工作，加快创业孵化基地建设。统筹做好高校毕业生、退役军人、城镇就业困难人员、农村转移劳动力等各类群体的就业工作，实现城镇新增就业6.1万人、农村劳动力新增转移就业6.5万人次。

　　健全可持续社会保障体系。落实全民参保计划。加快大病保险、付费方式改革。完善企业职工基本养老保险政策，加强社会保险扩面征缴工作。加强“智慧人社”建设，全面做好社会保障“一卡通”工作。推进“智慧民政”建设，完善社会救助体系，提高城乡低保补助水平，全面落实农村五保供养政策，保障困难群众基本生活。深化收入分配制度改革，实施城乡居民收入倍增计划，深化机关事业单位工资和津补贴制度改革。

　　加快发展社会事业。继续实施教育发展“十大工程”，新建幼儿园、中小学校36所，加强乡村教师队伍建设。推进靖江王府及王陵、甑皮岩两个国家考古遗址公园和“红色文化”设施建设，继续支持灵渠申遗，全面完成村级公共服务中心、示范（数字）农家书屋建设任务，提升百姓大舞台、百姓大讲坛、漓江之声、读书月等文化惠民特色品牌内涵，持续抓好“扫黄打非”工作。加强新型智库建设。加快医疗卫生体制改革，继续巩固和完善新农合制度，探索以医联体为基础的分级诊疗体系建设，实施居民健康卡服务，加强医疗系统信息化建设，大力发展中医药事业。继续做好人口计生工作，实施全面两孩政策。完善公共体育服务体系，深入开展全民健身活动。

　　加强和创新社会治理。继续做好普法工作。健全“三社联动”机制，提高城市治理的社会参与度。完善突发事件应急处置机制，加大敏感时期、重点时期的维稳监控力度，加强社区矫正工作，妥善处置群体性事件。加强对‘黄、赌、毒’违法犯罪的打击力度，及时排除各种安全隐患和漏洞。完善人民调解机制，依法规范信访秩序。强化安全生产责任，加强公共安全管理，强化食品药品和农产品质量安全监管。

　　加强国防后备力量建设，大力开展双拥共建活动，推进军民深度融合，支持工、青、妇等人民团体发挥桥梁纽带作用，发挥民主党派参政议政作用，做好民族宗教、外事侨务、人民防空、气象地震、防灾减灾、决策咨询、史志档案等工作，促进各项事业协调发展。

　　继续实施为民办实事工程。在全面完成自治区下达我市重点民生项目任务基础上，筹措资金60亿元，重点抓好生态、交通、教育、扶贫、新农村、社区、旅游设施等一批惠民项目。

　　各位代表！做好今年和“十三五”经济社会发展各项工作，必须进一步加强政府自身建设，努力提高新常态下推动发展的能力和水平。依法全面履行政府职能，严格按照法定权限和法定程序履行责任、行使权力，严格规范公正文明执法。健全依法决策机制，认真执行重大行政决策合法性论证程序，推进政府立法工作。全面深化政务公开，加强电子政务平台建设，提高政府公信力和执行力。加强权力监督制约，自觉接受人民代表大会及其常委会的法律监督、工作监督，接受人民政协的民主监督，接受司法机关、新闻舆论和社会公众的监督，认真执行人大及其常委会的决定、决议，办理代表建议和政协提案。扎实推进政府协商，认真听取各民主党派、工商联、无党派人士和人民团体的意见建议。巩固“三严三实”专题教育成果，持续开展反“四风”和反腐败专项整治活动，健全政府系统惩治和预防腐败体系，进一步改进作风，形成长效机制。严格落实党风廉政建设主体责任，履行“一岗双责”，建设高效、廉洁、法治政府。

　　各位代表！“十三五”和今年经济社会发展任务十分繁重，责任十分重大。让我们紧密团结在以习近平同志为总书记的党中央周围，在自治区党委、政府和市委的坚强领导下，凝心聚力，扎实工作，为全面实现“两个建成”目标、谱写桂林人民美好生活新篇章而努力奋斗！
